# Autoimmune Channelopathies of the Nervous System

**DOI:** 10.2174/157015911796557966

**Published:** 2011-09

**Authors:** Kleopas A Kleopa

**Affiliations:** Neurology Clinics and Neuroscience Laboratory, The Cyprus Institute of Neurology and Genetics, Cyprus

**Keywords:** Ion channels, autoimmunity, voltage-gated calcium channels, voltage gated potassium channels, glutamate receptors, limbic encephalitis, Morvan’s syndrome, Lambert-Eaton myasthenic syndrome, neuromyotonia.

## Abstract

Ion channels are complex transmembrane proteins that orchestrate the electrical signals necessary for normal function of excitable tissues, including the central nervous system, peripheral nerve, and both skeletal and cardiac muscle. Progress in molecular biology has allowed cloning and expression of genes that encode channel proteins, while comparable advances in biophysics, including patch-clamp electrophysiology and related techniques, have made the functional assessment of expressed proteins at the level of single channel molecules possible. The role of ion channel defects in the pathogenesis of numerous disorders has become increasingly apparent over the last two decades. Neurological channelopathies are frequently genetically determined but may also be acquired through autoimmune mechanisms. All of these autoimmune conditions can arise as paraneoplastic syndromes or independent from malignancies. The pathogenicity of autoantibodies to ion channels has been demonstrated in most of these conditions, and patients may respond well to immunotherapies that reduce the levels of the pathogenic autoantibodies. Autoimmune channelopathies may have a good prognosis, especially if diagnosed and treated early, and if they are non-paraneoplastic. This review focuses on clinical, pathophysiologic and therapeutic aspects of autoimmune ion channel disorders of the nervous system.

## 1 INTRODUCTION

An emerging group of neurological disorders is associated with autoantibodies acting on ligand-gated ion channels (receptors) or on voltage-gated ion channels. These disorders can be divided into those in which the antibodies are markers for an inflammatory process targeting neurons but themselves likely not pathogenic (including anti-Hu and anti-Yo antibodies) [1, 2], and those in which the antibodies are directly pathogenic. This distinction is important since in the former the usefulness of immunotherapies is limited [3, 4], whereas the latter group of diseases typically responds better to treatment [5, 6]. In the first category the neurological disorder is thought to be caused by a T cell mediated attack on the neurons expressing the antigen, and the associated inflammation that this produces. The autoantibodies indicate that the patient has a tumor, but they are not usually directly pathogenic as the antigenic target is often intracellular. In the second category, to which autoimmune channelopathies belong, there is strong evidence that antibodies are directly pathogenic by reducing the expression and function of ion channels at the cell membrane. Their pathogenicity has been shown by passive transfer of purified IgG to mice [7], and by application to neuronal cell line cultures or transfected cells *in vitro* [8-10]. These autoimmune channelopathies can be diagnosed by standard laboratory tests and usually respond to immunotherapies that reduce the levels of the pathogenic autoantibodies [11].

Autoimmune channelopathies can occur with or without cancer [12]. Paraneoplastic conditions are defined as those in which the neurological disease is secondary to the presence of a tumor, but is not directly caused by the tumor itself. They result from the immune response that is generated against the tumor, which can usually be shown to express the neuronal antigen; the resulting T cells and autoantibodies cross-react with neuronal tissue [13]. Phenotypically, paraneoplastic cases may not differ from non-paraneoplastic ones, although in some entities subtle clinical differences are being recognized. Certain channelopathies are associated with a higher risk of a paraneoplastic cause compared to others.

## 2 DISORDERS ASSOCIATED WITH AUTOIMMUNITY TO VOLTAGE GATED CALCIUM CHANNELS (VGCC)

### 2.1 Lambert Eaton Myasthenic Syndrome (LEMS)

Lambert-Eaton myasthenic syndrome (LEMS) [14] is a prototype paraneoplastic neurological syndrome: up to 60% of cases are associated with small cell lung cancer (SCLC) and rarely with other tumors [15]. Paraneoplastic LEMS may coexist with paraneoplastic encephalomyelitis, cerebellar degeneration (below), and sensory neuronopathy, especially in cases with SCLC [16]. Non-paraneoplastic cases tend to have a slower symptom progression and associate with other autoimmune conditions [15]. LEMS usually precedes tumor diagnosis by several months to years, presenting with progressive proximal weakness, affecting the legs more than the arms. There is less fatigability than in myasthenia gravis, but often muscle stiffness or pain after exertion. LEMS also differs from myasthenia in that it affects trunk and limb muscles rather than bulbar ones. Respiratory and craniobulbar involvement is uncommon in LEMS and is generally mild when present [15]. Ptosis is not uncommon, but ophthalmoplegia should raise suspicion for superimposed encephalitis [16]. Autonomic dysfunction is prominent with dry mouth, dry eyes, impotence, orthostatic hypotension, and hyperhidrosis. Neurological examination shows proximal weakness and waddling gait, with characteristic improvement in muscle power after brief maximal voluntary muscle activity. Reflexes are diminished but may improve after forceful exercise of the corresponding muscles.

LEMS is a presynaptic disorder of neuromuscular transmission. The autoimmune process results in depletion of VGCC and reduced calcium influx into the nerve terminal, leading to impaired quantal release of acetylcholine at the neuromuscular junction (NMJ). Acetylcholine release increases with high frequency nerve stimulation or by raising extracellular calcium [17, 18]. Freeze-fracture studies in LEMS showed that the active zone particles on the presynaptic membrane, generally considered to be the VGCC, were reduced in numbers and formed into small aggregates rather than into the normal double parallel arrays [19]. The disease including the electrophysiological features could be passively transferred to mice by daily injection of IgG for three or more days [20]. These observations along with the demonstration that the majority of patients responded well to immunomodulatory clearly implicated VGCCs as a target antigen in LEMS [21].

**Fig. (1):**
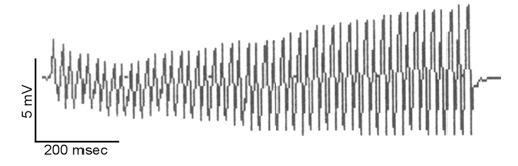
Electrophysiologic findings in Lambert-Eaton myasthenic syndrome. Repetitive stimulation of the ulnar nerve (50 Hz, train of 50 stimulations) and recording at the abductor digiti minimi muscle in a patient with LEMS shows a ~100% increase in the compound muscle action potential amplitude (from 2.09 mV at the beginning to 4.04 mV at the end of the stimulation), characteristic of presynaptic neuromuscular junction dysfunction.

Nerve conduction studies in patients with LEMS typically show reduced compound muscle action potential (CMAP) amplitudes recorded from rested muscle, which increase by at least 100% or more after brief voluntary isometric contraction [22]. A similar CMAP increment occurs with high-frequency repetitive stimulation of 20-50 Hz (Fig. **1**), that may be used when patients are unable to voluntarily exercise. The CMAP increment likely results from facilitation of acetylcholine release mediated by calcium influx. In contrast, routine low-frequency stimulation (2 to 3 Hz) results in decremental motor response, as in myasthenia. Needle electromyography (EMG) shows variability in amplitude and configuration of the recruited motor unit potentials (MUP). When neuromuscular dysfunction is severe, MUP may appear myopathic due to complete block of transmission to individual muscle fibers within each motor unit [15]. Single fiber EMG shows very high jitter values and blocking, both of which decrease as the MUP firing frequency increases, in contrast to myasthenia.

VGCC are a family of multimeric ion channels that participate in the generation of action potentials in dendrites, initiation of neurotransmitter release, and a variety of intracellular regulatory processes. Different subtypes associated with specific genes, designated L, N, P/Q, R, or T differ in their voltage dependence, inactivation rate, ionic selectivity, and pharmacology [23]. Each Ca^2+^ channel is composed of several different subunits (α1, α2/δ, β and γ). The diversity of genes encoding α1 subunits produces Ca^2+^ channels with different properties and cell-specific expression. The large α1 subunit contains the voltage sensing and ion selectivity elements, forms the channel pore and determines its sensitivity to various neurotoxins allowing specific labeling of VGCC subtypes. Antibodies were first detected in 40%–50% of LEMS patients using ^125^I-conotoxin GVIA (^125^I-CgTx) from *Conus geographus* [24] to label N-type VGCCs extracted in digitonin from human brain tissue, although these are not thought to be functionally effective [25]. Subsequently, using ^125^I-conotoxin MVIIC (^125^I-CmTx) from *C. magus,* antibodies to mammalian P/Q-type VGCCs were demonstrated in over 85% of LEMS patients [21, 26]. Ca^2+^ influx through these channels is inhibited by incubation in IgG from LEMS patients [27]. The P/Q-type VGCC antibodies are present in patients with or without SCLC and SCLC cells express functionally active VGCC on their surface. The immune response to the tumor presumably leads to antibodies directed at these VGCCs which then cross-react with those at the NMJ to cause the neurological disease.

The most important aspect of LEMS management is the early identification and treatment of the underlying malignancy, which may also improve the neuromuscular manifestations. Several studies have searched for possible serological markers that may distinguish between paraneoplastic and non-paraneoplastic LEMS. Studies using recombinant proteins corresponding to the extracellular S5-S6 linker region of individual domains forming the α1 subunit of P/Q-type VGCC showed that an antibody response to domain IV is more common in LEMS without tumor than in paraneoplastic LEMS. This may have implications for diagnostic workup in LEMS patients without previously established diagnosis of a tumor. Additionally this could point towards a differential autoimmune pathogenesis between paraneoplastic and non-paraneoplastic LEMS [28]. Another possible marker for paraneoplastic LEMS is the presence of SOX1 antibodies: in a large series they were present in 64% of patients with LEMS and SCLC but in none with idiopathic LEMS [29]. However, routine measurement of VGCC antibodies in patients without clinical LEMS is unlikely to assist either in management of SCLC or in assessment of prognosis: 8% of SCLC patients may have raised serum VGCC antibodies but only 3% have LEMS on clinical and electrophysiological ground and VGCC antibody titers do not predict survival [30].

Symptomatic therapy for LEMS includes 3,4-diaminopyridine (3,4-DAP) and pyridostigmine. 3,4-DAP inhibits presynaptic voltage gated potassium channels (VGKC) and improves acetylcholine release. It has a narrow therapeutic window and continued monitoring during treatment is needed. Common side effects with doses of 20-80mg daily include perioral and acral paresthesias, and rarely palpitations and seizures [31]. Patients with aggressive disease may need immunosuppressive treatment, including corticosteroids, azathioprine, plasma exchange, and intravenous immunoglobulin (IVIg) [32]. Favourable response to rituximab has also been reported in a patient with LEMS and cerebellar syndrome associated with anti-VGCC antibodies [33]. 

### 2.2 Cerebellar Ataxia Associated with VGCC Antibodies 

Cerebellar dysfunction is one of the most common paraneoplastic neurological disorders [13]. The underlying malignancy is usually SCLC, gynaecological and breast tumours, and Hodgkin's lymphoma [4, 34]. Clinically, patients may present initially with viral-like illness, dizziness, nausea, or vomiting, followed by gait unsteadiness. Neurological dysfunction progresses rapidly into ataxia, diplopia, dysarthria, and dysphagia [35]. MRI may be normal initially, but in later stages cerebellar atrophy becomes apparent. Pathologically, the hallmark of paraneoplastic cerebellar degeneration (PCD) is an extensive loss of Purkinje cells that might be associated with inflammatory infiltrates in the cerebellar cortex, deep cerebellar nuclei, and inferior olivary nuclei. Inflammation however is less prominent in PCD associated with anti-VGCC compared to the anti-Hu positive cases [34].

Multiple autoantigens have been associated with PCD, including anti-Hu and anti-Yo antibodies. In a subset of patients without these antineuronal antibodies, raised VGCC antibodies are found. A small proportion of patients with paraneoplastic LEMS and associated SCLC also present with cerebellar ataxia. In a study of 39 patients with PCD, about 40% had raised levels of VGCC antibodies in their serum and CSF, with evidence of intrathecal antibody production in some. Less than half of these patients with positive VGCC antibodies had LEMS, suggesting that VGCC antibodies should be looked for in patients with PCD, even if there are no symptoms of LEMS [36]. In a bigger series of 57 patients with presenting symptoms of PCD, 20% of cases with raised P/Q-type VGCC antibodies had no clinical evidence of LEMS [34]. Compared to patients with PCD and P/Q-type VGCC antibodies, patients with PCD and positive anti-Hu antibodies were more likely to be female, to have multifocal neurological involvement, to be severely disabled, and to die from neurological causes [34]. In general, SCLC patients with PCD have a worse outcome than patients without this complication of cancer.

As in the case of LEMS, there is increasing evidence that antibodies to VGCC play a pathogenic role in PCD. In contrast to the antibodies that target intracellular antigens, antibodies against VGCC react with cell-surface epitopes; this and the fact that some patients have intrathecal synthesis of such antibodies has suggested a direct pathogenic role in the cerebellar dysfunction. The cerebellum, particularly the Purkinje and granule cells, are rich in P/Q and N-type of the VGCCs, which are also found at the NMJ and on the surface of SCLCs [37, 38]. Mice deficient in the gene encoding the P/Q-type VGCC develop ataxia and dystonia [39]. Autoantibodies against the P/Q-type VGCC subtype from LEMS patients have been shown to decrease the calcium currents in cultured rat Purkinje and granule cells [37]. Furthermore, in autopsy material, P/Q-type VGCC were markedly reduced in the molecular layer of the cerebellum of LEMS patients with PCD compared to controls [40]. More recently, using a polyclonal peptide antibody against a major immunogenic region in P/Q-type VGCCs, Liao *et al*. [41] demonstrated that such antibody was sufficient to inhibit the function of neuronal and recombinant VGCCs, alter cerebellar synaptic transmission, and confer the phenotype of cerebellar ataxia.

Despite the fact that VGCC antibodies are considered pathogenic, PCD patients, in contrast to patients with LEMS do not respond favorably to immunotherapy. This is most likely due to the degree of irreversible neuronal destruction. Treatment of the malignancy is needed for PCD stabilisation or symptom improvement with or without immunotherapy. The use of corticosteroids, plasma exchange, IVIg, cyclophosphamide, and tacrolimus had no significant impact on neurological outcome of patients whose tumours were successfully treated [13, 34].

## 3 DISORDERS ASSOCIATED WITH ANTIBODIES TO VOLTAGE-GATED POTASSIUM CHANNELS (VGKC)

### 3.1 Acquired Neuromyotonia

There is a spectrum of disorders that arise from peripheral nerve hyperexcitability including neuromyotonia and cramp-fasciculation syndrome [42]. They can be inherited [43] or acquired on autoimmune basis. Acquired neuromyotonia typically presents with muscle fasciculations and painful cramps, stiffness, myokymia, pseudomyotonia (delayed relaxation of contracted muscles) and hyperhydrosis. The limb and trunk muscles are most commonly affected. Up to 20% of patients also have sensory symptoms such as paraesthesias, suggesting that sensory as well as motor nerves may be hyperexcitable [44].

EMG in patients with neuromyotonia reveals spontaneous and repetitive (doublet, multiplet) discharges of single MUPs with variably high intraburst frequency. This neuromyotonic activity can always be blocked by local curare and variably by nerve block [45], indicating that it originates in the motor nerve rather than in the muscle itself. There is evidence that the most distal portion of the nerve close to the NMJ is where hyperexcitability usually develops [46, 47]. Cramp-fasciculation syndrome is probably a milder version of the same condition, without neuromyotonic discharges in the EMG [42].

Neuromyotonia can be associated with other autoimmune diseases including myasthenia, rheumatoid disease, systemic lupus erythematosus, diabetes mellitus, and others [45]. Acquired neuromyotonia can also be a paraneoplastic condition, associated with thymoma in 10%–20% of cases, and more rarely with SCLC, Hodgkin’s lymphoma, and other malignancies. 

**Fig. (2):**
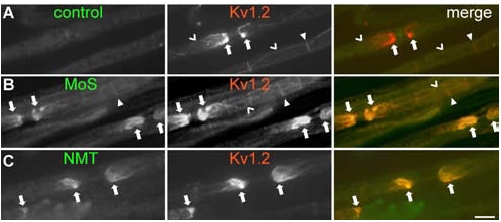
**Binding of sera from patients with neuromyotonia (NMT) or Morvan’s Syndrome (MoS) to juxtaparanodal areas.** Images of fixed mouse sciatic nerve teased fibers double stained with patient sera as indicated or a control serum (green) and a commercial antibody against Kv1.2, show colocalization of the MoS and NMT sera, but not of the control serum, with Kv1.2 expressed at axonal juxtaparanodes (arrows) as well as at the juxtaincisures (open arrowheads). Scale bar: 10 µm.

Immunoprecipitation of ^125^I-dendrotoxin-VGKCs extracted from human or rabbit cortex, demonstrated antibodies to VGKCs in about 40% of patients with NMT, although the antibody levels are not very high, and the test is less robust than that for acetylcholine receptor (AchR) or VGCCs [8]. Dendrotoxin only binds to the Kv1.1, 1.2 and 1.6 members of the *Shaker*-like potassium channels. The sensitivity of detecting VGKC antibodies has been increased by demonstrating binding of patient sera to relevant neural tissues with high expression of VGKC (Fig.**2**), as well as to different Kv subunits expressed in transfected *Xenopus* oocytes or in mammalian cells [48, 49].

To clarify the mechanism underlying NMT, passive transfer of IgG antibodies to mice resulted in resistance to curare [7], a modest increase in quantal content with prolonged action potentials in sensory nerves, and repetitive activity in dorsal root ganglion cultures [8]. These effects resemble the ones obtained with low concentrations (50 μM) of the potassium channel blockers 3,4-diaminopyridine or 4-aminopyridine. NMT patients may improve with plasma exchange and other immunosuppressive treatments. Symptomatic relief may be achieved with antiepileptic medications, which reduce nerve excitability by blocking sodium channels [50].

### 3.2 Morvan’s Syndrome

Morvan’s syndrome (MoS) or Morvan’s fibrillary chorea [51] describes rare patients with a combination of neuromyotonia and CNS involvement. MoS can be associated with heavy metal intoxication (gold, mercury) or other toxins and also thymomas, suggesting an autoimmune etiology. Clinically, the patients present with a combination of peripheral hyperexcitability with autonomic dysfunction such as hyperhidrosis and hypersalivation, as well as CNS disturbance, in the form of severe insomnia, mental confusion, visual hallucinations, delirium and prominent circadian hormonal disturbances [52-54].

VGKC antibodies can be modestly or highly raised in most MoS patients. In general patients either improve spontaneously over time or respond more quickly to immunosuppressive treatments including steroids, azathioprine, IVIg, and particularly plasma exchange [52, 53, 55-57] or immunoadsorption [58].

### 3.3 Limbic Encephalitis

Limbic encephalitis (LE) has been recognized as an autoimmune, non-paraneoplastic VGKC antibody-mediated disease affecting exclusively the CNS and most prominently the limbic areas [5, 59]. Patients develop acutely or subacutely, memory loss and confusion, leading over days or weeks to epileptic seizures (either temporal lobe or partial complex seizures). Cognitive impairment is profound with substantial retrograde and anterograde memory loss. MRI shows high signal in the hippocampal regions. In contrast to paraneoplastic LE, VGKC-antibody associated LE cases do not usually have a tumor, and can respond well, albeit sometimes over weeks rather than days, to immunotherapies [5, 6] with good recovery of memory and other CNS functions. REM sleep disturbances and hyponatraemia are common in both LE and MoS [60] and some patients develop hypothermia, hypersalivation, pain, and disorders of appetite [61]. In contrast to patients with MoS, LE patients lack clinical manifestations of peripheral nerve hyperexcitability and most have normal peripheral electrophysiological studies as well [5].

Patients with paraneoplastic LE usually have a SCLC and in around 50% anti-Hu antibodies are detectable. Other associated onconeuronal antibodies in LE include Ma2, CV2/CRMP5 or amphiphysin [13]. Although most LE cases with VGKC antibodies are non-paraneoplastic, in up to 30% of cases there is also an underlying malignancy [62]. The neurological prognosis seems worse in VGKC antibody positive patients with paraneoplastic LE [63]. The presence of additional antibodies to anti-glial nuclear antibodies may predict paraneoplastic LE in patients with VGKC-antibodies [64]. Compared with other paraneoplastic or immune-mediated LE, the CSF of patients with antibodies against VGKCs shows less pleocytosis and lower protein concentration and intrathecal synthesis of IgG [65, 66].

Further paraneoplastic LE forms are associated with antibodies to hippocampal neuropil, which are located on the membrane with usually good prognosis after tumor removal [65]. A high proportion of patients with non-paraneoplastic LE without neuropil or VGKC antibodies have antibodies to a novel cell-membrane antigen [67]. Given the emerging complexity of immunological associations in LE, it appears useful to classify patients into two main subgroups according to the location of the target antigens, either on the cell surface, or intracellular. Overall, the former subgroup to which also cases with VGKC-antibodies belong tends to have a better prognosis, compared to the subgroup with intracellular target antigens [67]. Thus, about 80% of LE patients respond to corticosteroids, plasma exchange, or IVIg, especially if treated early, and if they have antibodies to cell surface antigens, including VGKC [5, 6, 67].

### 3.4 VGKC Antibodies in Epilepsy and Hyperekplexia

In addition to the disorders described above, VGKC antibodies were also found in 6% of patients with long-standing epilepsy [68], in one patient with hyperekplexia [69] and in some people without neurological symptoms. It is not surprising that autoimmunity to VGKCs may cause epilepsy since genetic mutations affecting different VGKC are associated with seizure disorders [70, 71]. Furthermore, Kv1.1-deficient mice develop spontaneous seizures with hippocampal excitability [72]. Many of these VGKC-antibody-positive patients have drug resistant epilepsy and complex partial seizures of temporal lobe origin. The highest titres of VGKC antibodies were found in older patients with acute or subacute onset of encephalopathic disorders. Immunomodulatory treatment resulted in reduction of seizures in some of these patients. Lower levels of VGKC antibodies were also found in cohorts of drug-resistant epilepsy patients, but immunotherapy was not instituted in these groups [68, 73]. Whether VGKC antibodies are pathogenic in these chronic epilepsy cases remains to be determined.

### 3.5 VGKC and Autoimmune Mechanisms 

VGKC are the most ubiquitous and diverse group of voltage-dependent ion channels found in the body [74]. Functional Kv channels are formed by homotetramers or heterotetramers of four alpha subunits, often associated with an auxiliary beta subunit [75]. The latter can modify the gating properties of the heteromultimeric channel complex [76]. This leads to a high degree of diversity in the neuronal K^+^ channel population, probably serving specific physiological requirements of different locations [74, 77]. VGKC are expressed widely in the CNS and PNS but show a highly specific localization of individual Kv1 subunits in each area that may be functionally important. Kv1.1 and Kv1.2 are co-expressed at the juxtaparanodal regions of the axons surrounding the nodes of Ranvier [78, 79]. Here, they account for the K_i_ current that can be recorded by patch clamping of myelinated axons [80, 81] and contribute to the regulation of axonal membrane excitability, preventing repetitive discharges. 

In the CNS, a more complex distribution of numerous VGKC subunits in different regions has emerged [82], but the cerebellum and hippocampus have received most attention due to the genetic disorders associated with dysfunction of VGKC in these areas, especially epilepsy and episodic ataxia [71, 83]. Kv1.1-1.6 are highly expressed in different combinations in the cerebellar molecular and granule cell layers as well as at the basket cell terminals [84]; and in specific layers of the hippocampus that contain excitatory axon terminals, most prominently in the molecular layer of the dentate gyrus, in the CA3 mossy fiber zone and in the stratum radiatum of CA1-3 [85-87]. VGKC play a major role in regulating the excitability of hippocampal neurons by modulating neurotransmitter release, postsynaptic responses to excitatory inputs, neuronal spike properties, and firing frequency [88].

How autoantibodies impair VGKC function remains unclear. They appear to be heterogeneous and may bind to multiple Kv subunits [48, 49]. VGKC antibodies in NMT do not appear to block the function of *Shaker*-type K^+^ channels directly, since short incubation (up to 2 hr) of cultured neuronal cell lines with IgG from NMT patients has no effect. However, when applied for several hours, neuromyotonia antibodies reduce K^+^ channel current amplitudes without changing gating kinetics [89, 90]. Thus, these antibodies appear, in a mechanism independent of added complement, to accelerate channel turnover and degradation of VGKC [9].

**Fig. (3):**
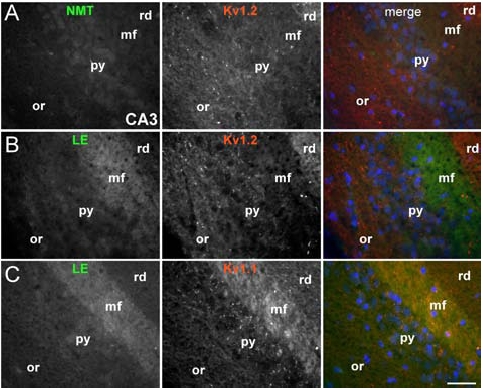
**Distinct binding of limbic encephalitis (LE) sera to hippocampal areas expressing VGKC.** These are images of sections from fixed mouse hippocampus (CA3 area) immunostained with sera from NMT or LE patients as indicated (green) and commercial antibodies against Kv1.2 (A-B) or Kv1.1 (C) (red). LE sera (but not the NMT serum) bind strongly to the mossy fiber layer (mf) where Kv1.1 but not Kv1.2 is prominently expressed. Kv1.2 is more prominent in the radiatum (rd) but LE sera do not bind there (or: stratum oriens; py: pyramidal layer).

The question why certain patients with VGKC antibodies present with a CNS disorder but others have only symptoms of peripheral nerve hyperexcitability, remains unresolved. Recent evidence suggests that sera from NMT, MoS LE patients may differentially affect the VGKC subtypes. Immunohistochemically, serum from VGKC-positive patients has been shown to produce distinct staining patterns on mammalian hippocampus similar to those observed by specific anti-Kv1 antibodies For example, serum from LE patients binds more prominently to Kv1.1-enriched areas of the hippocampus [49] (Fig. **3**). It remains to be shown whether the distinct phenotypes of VGKC-associated disorders are produced by differential specificity of antibodies for VGKC subtypes or associated proteins that are distinctly localized in the CNS and PNS.

## 4 ALPHA3 GANGLIONIC ACETYLCHOLINE RECEPTOR ANTIBODIES IN AUTOIMMUNE AUTONOMIC NEUROPATHIES

Autoimmune aetiology has been recognized in some forms of peripheral autonomic disturbance. Autoimmune dysautonomias develop over days or weeks, persist for some months and then may partially resolve. Both the sympathetic and parasympathetic systems may be involved. Patients present with orthostatic hypotension, impaired sweating, dry mouth and eyes, sexual dysfunction, urinary retention, impaired pupillary responses and defects in heart rate control. Gastrointestinal disturbances such as constipation or diarrhea may also occur. In a proportion of patients raised antibodies to the (α3-type) acetylcholine receptor (AchR) containing the α3 and β4 subunits, that is present in ganglionic synapses of the autonomic system, were detected [91]. Other cases may be associated with paraneoplastic antibodies such as anti-Hu and CRMP-5 [92]. Patients with orthostatic hypotension and prominent cholinergic dysautonomia are most likely to be seropositive for the ganglionic AchR antibody [93].

Evidence for the pathogenicity of α3 AchR antibodies comes from studies showing correlation between titres and degree of autonomic impairment not only within an individual patient but also between patients. An animal model of the disease in the rabbit recapitulates the important clinical features of the human disease [94]. Furthermore, a single injection of the rabbit or human antibodies into mice leads to autonomic dysfunction which may last for a few days and is followed by a compensatory overshoot in function as they recover [95]. Patients with chronic autoimmune autonomic neuropathy may respond to immunotherapies [96].

## 5 ANTIBODIES AGAINST GLUTAMATE AND GABA RECEPTORS

### 5.1 Encephalitis associated with NMDA receptor antibodies

The presence of antibodies against the N-methyl-D-aspartate (NMDA) glutamate receptor was initially descibed in young women presenting with prodromal symptoms including headache, fever, or a viral-like illness, and then develop severe psychiatric symptoms, memory loss, seizures, decreased consciousness, accompanied by dyskinesias, hypo-ventilation, or autonomic instability. The underlying malignancy was ovarian teratoma [97]. Further studies in larger series of patients confirmed that this is a disease affecting predominantly young women in over 90% of cases with median age of 23 years (range 5-76 years) with the typical clinical features as initially described [98]. In 60% of cases there was an underlying tumour, most commonly ovarian teratoma. Patients who received early tumour treatment and immunotherapy had better outcome. Immunotherapy often results in neurological improvement enabling tumour removal [99].

Autoimmunity involving NMDA receptors has recently been identified also in children and a few men without a tumour [100]. Some patients previously described as having acute diffuse lymphocytic meningoencephalitis, juvenile acute non-herpetic encephalitis, or acute juvenile female non-herpetic encephalitis had anti-NMDA receptor encephalitis [101]. Dyskinetic encephalitis lethargica in very young children has also been associated with anti-NMDA antibodies [102]. Further studies in children showed that 40% of all encephalitis cases are associated with anti-NMDA antibodies. Younger patients are less likely to have tumors, and clinically the phenotype resembles that of the adults, although dysautonomia and hypoventilation are less prominent in children [103]. As with other channelopathies discussed above, antibodies to NMDA receptors are also detected in a proportion of patients with unexplained new-onset epilepsy. These cases present with prominent psychiatric symptoms and CSF pleocytosis, and they may develop hypoventilation [104].

The main epitope targeted by the antibodies is in the extracellular N-terminal domain of the NR1 subunit. Patients' antibodies decreased the numbers of cell-surface NMDA receptors and NMDA-receptor clusters in postsynaptic dendrites, an effect that could be reversed by antibody removal, strongly indicating that anti-NMDA antibodies are pathogenic [98]. Anti-DNA antibodies, which cross-react with the NR2A and NR2B subunits of the NMDA receptor through a conserved pentapeptide sequence, were shown to cause hippocampal cell death when injected into mouse brains, and animals immunised with the NR2A peptide developed high titres of specific NR2a antibodies which were able to gain access to the hippocampus. These animals developed memory loss [105]. The antibodies may act by stimulation of the NMDA receptor leading to glutamate-induced neurotoxicity.

### 5.2 Encephalitis Associated with AMPA Receptor Antibodies 

Another glutamate receptor has been recently added to the list of antigens involved in LE. In a series of 10 patients, Lai *et al*. [106] discovered antibodies to GluR1 and GluR2 subunits of the alpha-amino-3-hydroxy-5-methyl-4-isoxazolepropionic acid (AMPA) glutamate receptor. Median age was 60 (38-87) years. The majority were women and most of them had tumors of the lung, breast, or thymus. Most patients responded to immunotherapy or oncological therapy, but neurological relapses, without tumor recurrence, were frequent and influenced the long-term outcome. When applied to cultures of neurons, the patient’s antibodies significantly decreased the number of GluR2-containing AMPA receptor clusters at synapses and these effects were reversed after antibody removal, confirming their pathogenicity [106].

Autoimmunity to glutamate receptor 3 (GluR3), that also belongs to AMPA type, has long been associated with Rasmussen’s encephalitis, a severe form of intractable childhood epilepsy that is usually restricted to one hemisphere and leads to marked cognitive decline. The disease is associated with inflammatory and degenerative changes in the cerebral cortex and is extremely resistant to treatment. Immunization of rabbits with the GluR3 extracellular domain resulted in inflammation of the cortex and seizure activity [107]. However, the mechanism of action of these antibodies and their frequency and specificity in patients remains somewhat controversial, while there is accumulating evidence that Rasmussen’s encephalitis is a T cell mediated disorder [108] and GluR3 antibodies may be secondary to the neuronal damage. 

### 5.3 Epilepsy Associated with Antibodies to GABA Receptor

More recently, antibodies to the GABA(B) receptor have been reported in a series of patients presenting with early or prominent seizures and other symptoms, MRI, and electroencephalography findings consistent with predominant limbic dysfunction [109]. Immunoprecipitation and mass spectrometry showed that the autoantibodies recognize the B1 subunit of the GABA(B) receptor, an inhibitory receptor that has been associated with seizures and memory dysfunction when disrupted. Half of the cases were found to have tumours, mostly SCLC. Most patients responded to immunotherapy and cancer treatment.

## 6 AQUAPORIN-4 ANTIBODIES IN NEUROMYELITIS OPTICA

Neuromyelitis optica (NMO) or Devic’s syndrome is an inflammatory and necrotizing disease clinically characterized by selective involvement of the optic nerves and spinal cord [110]. Both monophasic and relapsing NMO have been known since the original description over hundred years ago. Bilateral optic nerves are severely affected and the myelitis is distinct, extending three or more spinal cord segments, and showing prominent cord cavitation and swelling. NMO predominantly affects young women and can be associated with other autoimmune diseases. Although NMO often follows a more aggressive clinical course than multiple sclerosis (MS) it may respond better to immunosuppressive therapy [111].

There has been a long controversy as to whether NMO is a variant of MS or a distinct disease. In contrast to other demyelinating diseases, pathological examination in autopsy cases with NMO showed vasculocentric IgM, IgG and complement component co-deposition [112]. Cellular infiltrates included predominantly neutrophils, eosinophils and B cells, with only a few T cells, suggesting humoral immunity as the major aetiology in NMO. Immunofluorescence demonstrated that NMO-IgG selectively binds to the pia mater, Virchow-Robin spaces and the abluminal surface of cerebral microvessels [113], where it co-localises with aquaporin-4 (AQP-4), a water channel protein, mainly expressed in astroglial foot processes. Subsequently, specific NMO-IgG binding was observed to cells expressing full length AQP-4, while it was absent in the brains of AQP-4-null mice [114].

An immunohistopathological study in post-mortem brain and spinal cord tissue revealed that AQP-4 immunoreactivity was lost in 90% of acute and chronic NMO lesions in contrast to demyelinated MS plaques. Thus, astrocytic impairment associated with the loss of AQP-4 and humoral immunity may be important in the pathogenesis of NMO lesions [115]. Recent studies have demonstrated that human anti-AQP-4 antibodies are not only important in the diagnosis of NMO but also pathogenic: they induced NMO-like lesions in animal models [116-118].

## 7 CONCLUSIONS

Autoimmune channelopathies have expanded rapidly in the last decades to include disorders of the peripheral, autonomic and central nervous systems and an increasing number of target antigens. Although some of the neurological manifestations have been known for over a century, we are now beginning to understand the immunological associations and molecular mechanisms that lead to ion channel dysfunction in neural tissues. Parallel progress in the discovery of mutations in ion channel genes causing phenotypically similar genetic disorders has stimulated further research into the common pathophysiological mechanisms. Acquired channelopathies are usually adult onset disorders some of which present relatively acutely or take a more subacute course. In most cases the underlying cause of the disease is not clear, but a tumor (especially SCLC or thymoma) must always be considered. The autoantibodies act mainly by increasing the turnover of the target antigens, depleting the neural tissue of their function. Some antibodies may bind to the agonist site or ion channel pore affecting the opening of the channel. Patients with autoimmune channelopathies may respond well to immunotherapies, and neurological damage may be minimized if diagnosis and treatment are instituted early. Therefore, it is important to recognize them clinically and understand fully the mechanisms of these disorders, in order to improve therapeutic possibilities and outcome.

## ABBREVIATIONS

LEMS: = Lambert-Eaton myasthenic syndrome
SCLC: = Small cell lung cancer
NMJ: = Neuromuscular junction
VGCC: = Voltage gated calcium channels
CMAP: = Compound muscle action potential
EMG: = Needle electromyography
MUP: = Motor unit potential
3,4-DAP: = 3,4-diaminopyridine
IVIg: = Intravenous immunoglobulin
PCD: = Paraneoplastic cerebellar degeneration
VGKC: = Voltage-gated potassium channels
AchR: = Acetylcholine receptor
MoS: = Morvan’s syndrome
LE: = Limbic encephalitis
NMDA: = N-methyl-D-aspartate
AMPA: = Alpha-amino-3-hydroxy-5-methyl-4-isoxazole-propionic acid
